# Cardiovascular medication in patients with raised NT-proBNP, but no heart failure in the SHEAF registry

**DOI:** 10.1136/openhrt-2022-001974

**Published:** 2022-06-01

**Authors:** Elena Wolodimeroff, Pankaj Garg, Andrew J Swift, Graham Fent, Nigel Lewis, Dominic Rogers, Athanasios Charalampopoulos, Abdallah Al-Mohammad

**Affiliations:** 1 Department of Infection, Immunity & Cardiovascular Disease, The University of Sheffield Medical School, Sheffield, UK; 2 Norwich Medical School, University of East Anglia, Norwich, UK; 3 Department of Cardiology, Sheffield Teaching Hospitals NHS Foundation Trust, Sheffield, UK

**Keywords:** heart failure, hypertension, echocardiography, atrial fibrillation

## Abstract

**Objectives:**

We aim to assess the association of cardiovascular medications with outcomes of patients referred to the diagnostic heart failure (HF) clinic with symptoms or signs of possible HF, raised N-terminal pro-brain-type natriuretic peptide (NT-proBNP) but no evidence of HF on transthoracic echocardiography (TTE).

**Methods:**

Data were collected prospectively into the Sheffield HEArt Failure (SHEAF) registry between April 2012 and January 2020. The inclusion criteria were symptoms or signs suggestive of HF, NT-proBNP >400 pg/mL, but no evidence of HF on TTE. Cox proportional-hazards regression model was used to investigate the association between the survival time of patients and different cardiovascular medications. The outcome was defined as all-cause mortality.

**Results:**

From the SHEAF registry, we identified 1766 patients with raised NT-proBNP with no evidence of HF on TTE. Survival was higher among the younger patients, and among those with hypertension or atrial fibrillation (AF). Mortality was increased with male gender, valvular heart disease and chronic kidney disease. Using univariate Cox proportional-hazards regression, the only cardiac therapeutic agent independently associated with all-cause mortality was beta-blocker (HR 0.86; 95% CI: 0.77 to 0.97; p=0.02). The use of beta-blockers was significantly higher in patients with AF (63% vs 39%, p<0.01) and hypertension (51% vs 42%, p<0.01). However, using multivariate Cox proportional-hazards regression to adjust for all variables associated with mortality, the influence of beta-blockers became non-significant (HR 0.96; 95% CI: 0.85 to 1.1, p=0.49).

**Conclusion:**

When all variables associated with mortality are considered, none of the cardiovascular agents are associated with the improved survival of patients with suspected HF, raised NT-proBNP but no HF on echocardiography.

**WHAT IS ALREADY KNOWN ON THIS TOPIC**

- Using the National Institute for health and Care Excellence guidelines for the diagnosis and management of chronic heart failure (HF), we identified a group of patients with raised N-terminal pro-brain-type natriuretic peptide (NT-proBNP) but no echocardiographic evidence of HF. These patients often have comorbidities that commonly occur in those with HF.

**WHAT THIS STUDY ADDS**

- Although simple assessment suggested possible association of beta-blockers with outcomes of patients referred to the diagnostic HF clinics with raised NT-proBNP and no echocardiographic evidence of HF, that observation was negated by adjusting for the covariates.

**HOW THIS STUDY MIGHT AFFECT RESEARCH, PRACTICE AND/OR POLICY**

- The absence of effect of the cardiovascular agents on outcomes of these patients should not dissuade one from using them when these patients have other clinical indications for their use in these patients.

## Introduction

The common symptoms and signs of heart failure (HF) are non-specific, necessitating the use of biomarkers such as natriuretic peptides (NPs) and imaging tests such as transthoracic echocardiography (TTE), as diagnostic tests. The rise in NP, such as B-type NP (BNP) and N-terminal pro-BNP (NT-proBNP), is a clinical biomarker of HF.^1^ Their use was recommended by the National Institute of health and Clinical Excellence (NICE) chronic HF guidelines (CG 108–2010).^2^ The latter’s update in 2018 (NG 106–2018) made NT-proBNP the gatekeeper into the diagnostic algorithm for patients suspected of HF in the community.^3^ Therefore, a patient with symptoms and/or signs raising suspicion of HF, whose NT-proBNP is ≥400 pg/mL, should be referred by primary care clinicians for specialist assessment and TTE.^4^ TTE can detect structural and functional cardiac abnormalities that define HF, its phenotype and sometimes its underlying cause.^5 6^ Patients thus referred to the HF specialist clinic with NT-proBNP ≥400 pg/mL and no diagnostic features of HF on TTE are deemed not to have HF. In a previous study, we demonstrated that these patients with no HF constitute 29% of all the patients referred to the HF clinic in Sheffield.^7^ These patients’ all‐cause mortality rate was 7.3 per 100 patient-years (95% CI 6.5 to 8.2) over a mean follow-up (FU) period of 6 years.^7^ The clinical variables independently associated with all-cause mortality in this group of patients were: age, male gender, chronic obstructive pulmonary disease, dementia and worsening chronic kidney disease (CKD) stage.^8^

The present study sought to investigate the association between the use of cardiovascular drugs and the risk of mortality among patients referred with symptoms or signs of HF, a raised NT-proBNP and no echocardiographic evidence of HF.

## Methods

### Data and patients

Patients suspected of HF whose NT-proBNP was ≥400 pg/mL were referred to the HF clinic in Sheffield in accordance with NICE guidelines.^2 3^ Data were collected prospectively and electronically encrypted in the Sheffield HEArt Failure (SHEAF) registry, with an annual data validation check. The analytical cohort was derived from all patients presenting to the HF clinic between 13 April 2012 and 31 January 2020. The inclusion criteria were: age 18 years or over, symptoms and/or signs suggestive of HF, raised NT-proBNP (≥400 pg/mL) but no evidence of HF on TTE.

### HF assessment

All patients underwent a resting 12-lead ECG and a TTE. The TTE in these patients had to show good biventricular contraction, no evidence of significant diastolic left ventricular dysfunction (E:e’ <13, left atrium (LA) <34 mL/m^2^, no evidence of left ventricular hypertrophy), no evidence of pulmonary hypertension and no severe valvular stenosis or regurgitation. Each patient was then clinically assessed by an HF specialist who concluded that there was no evidence of HF.

### Study variables

Clinical variables were obtained from the SHEAF registry. Data on whether the patient had atrial fibrillation or not were obtained from the 12-lead ECG. Details of the TTE findings were recorded in the database.

### Statistical analysis

Statistical analyses were performed in MedCalc V.19.0.5. Categorical baseline characteristics are described with numbers and percentages. Continuous variables are described using means and SD. Comparison of continuous variables was done using an independent t-test, while the comparison of categorical variables was done using Χ^2^ t-test. Where necessary, analysis of variance test was used to compare several groups. Kaplan-Meier curves were used to visualise and interpret the data of variables associated with mortality. Kaplan-Meier curves used the log-rank test to investigate the differences in curves between alive and dead patients at FU. Further survival analysis was done using the Cox proportional-hazards model to adjust for covariates.^9^ All tests were two sided, and statistical significance was considered if p value was <0.05.

## Results

### Demographics

From the SHEAF registry, we identified 1776 patients who presented to specialist HF clinics with suspected HF symptoms, raised NT-proBNP, but no evidence of HF on TTE. The mean age of the patients was 78±9 years old and 43% were male.

A total of 278 patients (16%) died at a mean FU period of 3 years. The demographic data and patients’ characteristics of both dead and alive patients are detailed in table 1. Compared with those who died, the patients who remained alive during FU were significantly younger (alive 78±9 vs dead 80±9, p<0.01), had significantly higher incidence of both hypertension (alive 64% vs 55%, p<0.01) and atrial fibrillation (alive 37% vs dead 22% p<0.01). Among those who died during FU, there were more male patients (alive 42% vs dead 49%, p=0.02) and more patients with valvular heart disease (alive 7% vs dead 10%, p=0.04). As expected, patients who have CKD II–V were significantly more likely to die during FU (alive 57% vs dead 68%, p<0.01). It was interesting, however, that no significant differences between the two groups with regard to history of diabetes mellitus, ischaemic heart disease, previous myocardial infarction, New York Heart Association functional class or NT-proBNP levels.

**Table 1:** Patients’ demographics and comorbidities

	Alive (n=1498)	Dead (n=278)	P value
Age (years)	78±9	80±9	<0.01
Male gender	627 (42%)	137 (49%)	0.02
NT-proBNP (pg/mL)	943±801	961±854	0.74
Hypertension	956 (64%)	154 (55%)	<0.01
Diabetes mellitus	250 (17%)	57 (21%)	0.12
Ischaemic heart disease	334 (22%)	72 (26%)	0.19
Valvular heart disease	99 (7%)	28 (10%)	0.04
Previous myocardial infarction	99 (7%)	26 (9%)	0.1
Atrial fibrillation	554 (37%)	60 (22%)	<0.01
Chronic kidney disease (II–V)	847 (57%)	190 (68%)	<0.01
New York Heart Association functional status	1.8±0.7	1.8±0.8	0.54

NT-proBNP, N-terminal pro-brain-type natriuretic peptide.

### Cardiovascular therapeutic agents

Table 2 shows the survival characteristics of patients with no HF on different cardiovascular therapeutic agents. Of the cohort, 848 patients (48%) were on beta-blockers. ACE inhibitors (ACEi) were used by 39% (n=697) of the cohort. The majority of the patients were not receiving loop (97%, n=1722) or thiazide diuretics (99%, n=1760). Mineralocorticoid receptor antagonists (MRAs) were used by 5% (n=84) of the patients, while digoxin was used in only 1% (n=14) of the patients.

**Table 2:** Cardiovascular therapeutic agents in both alive and dead cohorts during a mean follow-up period of 3 years

		Alive	Dead	P value
Beta-blocker	No therapy	768 (83%)	160 (17%)	0.05
	On therapy	730 (86%)	118 (14%)	
ACEi	No therapy	909 (84%)	170 (16%)	0.88
	On therapy	589 (84.5%)	108 (15.5%)	
MRA	No therapy	1431 (85%)	261 (15%)	0.24
	On therapy	67 (80%)	17 (20%)	
Loop diuretic	Dose decreased	1 (33%)	2 (67%)	0.037
	No therapy	1456 (85%)	266 (15%)	
	On therapy	41 (80%)	10 (20%)	
Thiazide diuretic	Dose decreased	3 (100%)	0 (0%)	0.756
	No therapy	1484 (84%)	276 (16%)	
	On therapy	11 (85%)	2 (15%)	
Digoxin	No therapy	1487 (84%)	275 (16%)	0.55
	On therapy	11 (79%)	3 (21%)	

ACEi, ACE inhibitor; MRA, mineralocorticoid receptor antagonist.

There was a trend for more patients on beta-blockers to be alive when compared with patients not on these agents (users: 86% vs non-users: 83% p=0.05) (table 2). There was a trend for those on a loop diuretic to fare better than those who were not on a loop diuretic or had their dose reduced (users: 86% vs non-users: 85% vs dose decreased: 33%, p=0.037). Other cardiovascular therapeutic agents, including ACEi, MRAs, thiazide diuretics and digoxin, did not have a statistically significant association with survival.

### Survival Cox proportional-hazards regression

**Figure 1:**
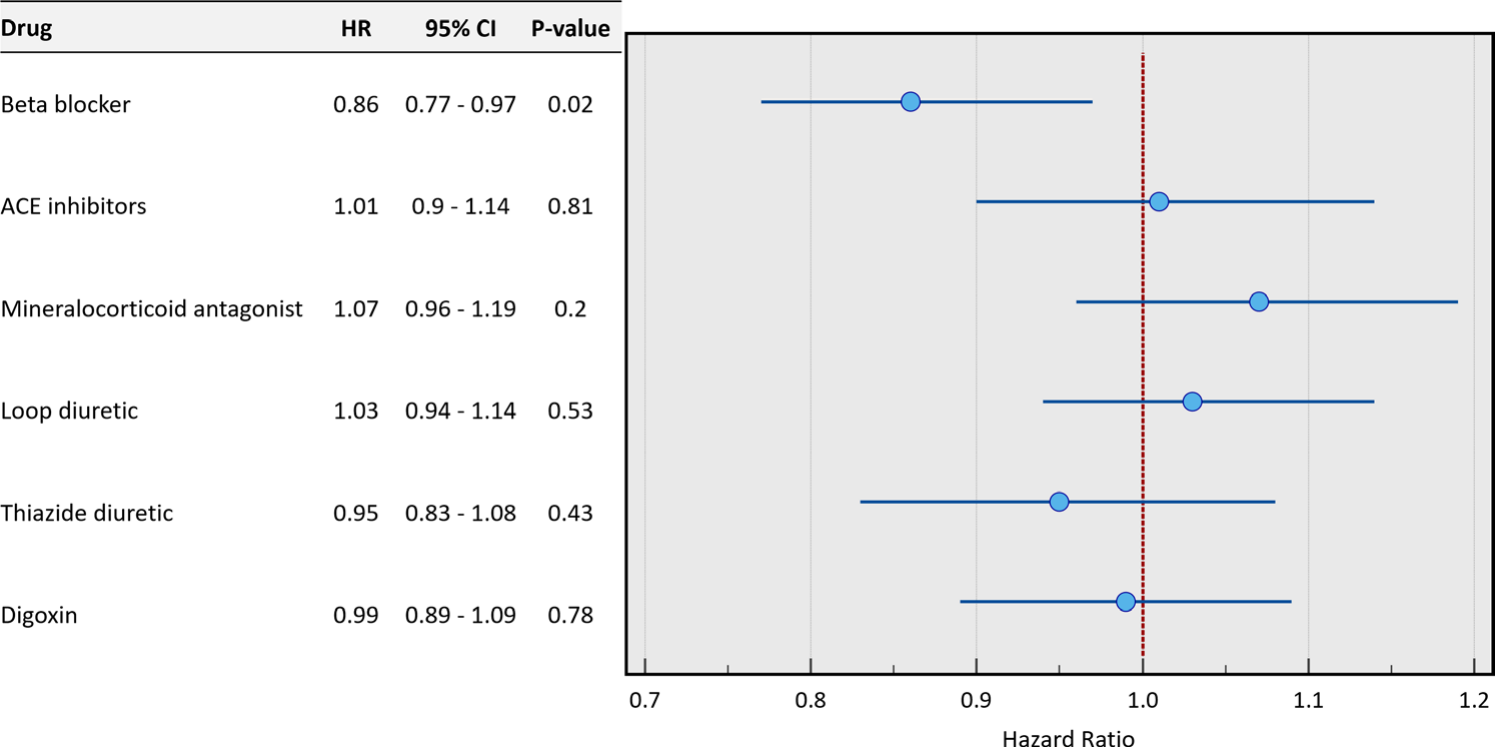
Forest plot of HRs of pharmacological therapy in patients with no heart failure. The lines represent the 95% CI.

Using univariate Cox proportional-hazards regression, from all the various cardiac therapeutic agents the patients were receiving, beta-blockers were the only cardiovascular therapy with independent association with all-cause mortality that just reached statistical significance (HR 0.86; 95% CI: 0.77 to 0.97, p=0.02) (figure 1).

However, using multivariate Cox proportional-hazards regression to adjust for all variables associated with mortality,^8^ the influence of beta-blockers became non-significant (HR 0.96; 95% CI: 0.85 to 1.1, p=0.49) (table 3).

**Table 3:** Multivariate Cox proportional-hazards regression of all variables associated with mortality

	Beta	HR	95% CI	P value
Age (years)	0.36	1.44	1.26 to 1.6	<0.0001
Beta-blocker	−0.04	0.96	0.85 to 1.1	0.4961
Gender (male)	0.21	1.24	1.1 to 1.4	0.0004
Hypertension	−0.14	0.87	0.78 to 1	0.0227
Valvular heart disease	0.04	1.04	0.94 to 1.2	0.3999
Chronic kidney disease	0.14	1.15	1.02 to 1.3	0.0277
Atrial fibrillation	−0.31	0.73	0.64 to 0.8	<0.0001

Age (HR 1.44; 95% CI: 1.26 to 1.64, p<0.0001) and male gender (HR 1.24; 95% CI: 1.10 to 1.39, p=0.0005) were independently associated with increased all-cause mortality in this cohort. Not surprisingly, CKD is associated with increased risk of mortality within the mean FU period of 3 years, though this impact just reached statistical significance (HR 1.15; 95% CI: 1.01 to 1.30, p=0.03). Two comorbidities appear on the other hand to be associated with reduced risk of all-cause mortality among these patients during FU. These were atrial fibrillation, which is significantly associated with lower all-cause mortality (HR 0.73; 95% CI: 0.64 to 0.84, p<0.0001), and hypertension, which was associated with less likelihood of death during the mean 3-year FU, although the impact of the latter only just reached statistical significance (HR 0.87; 95% CI: 0.78 to 0.98, p=0.0245).

### Kaplan-Meier survival curves

**Figure 2:**
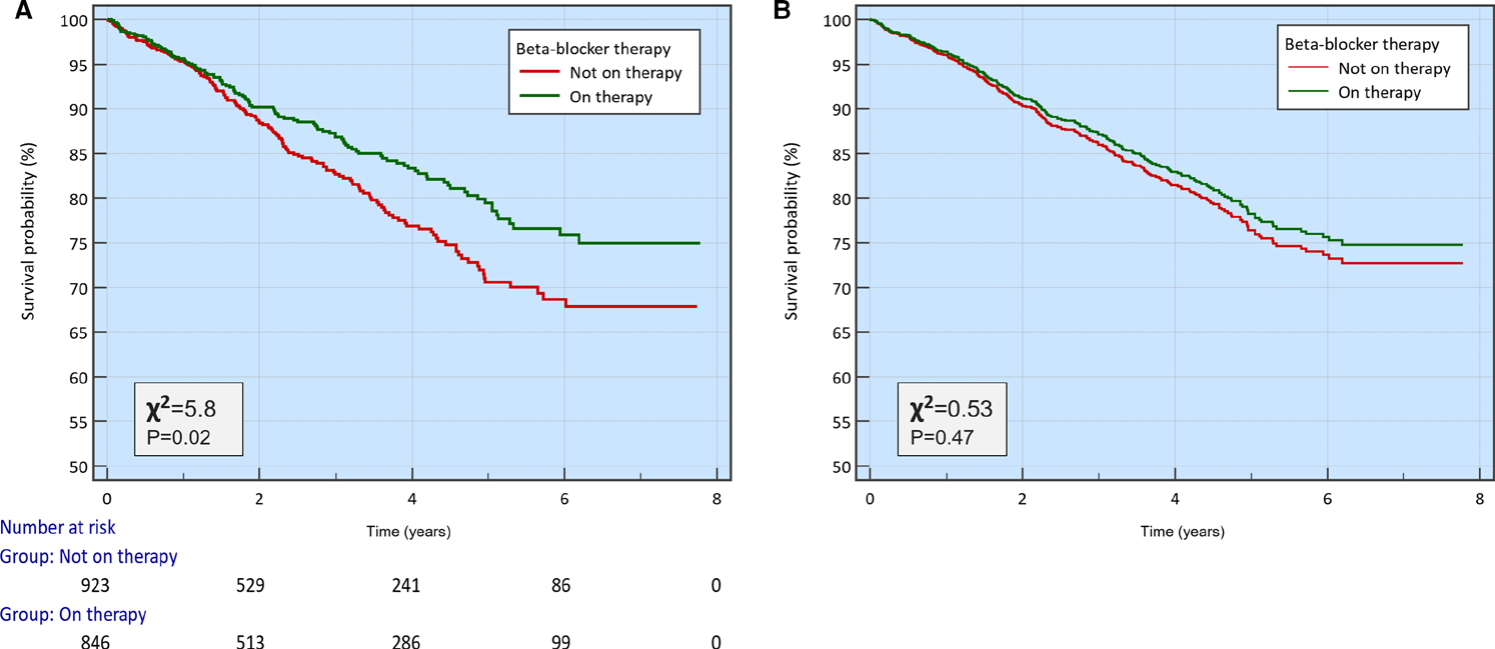
(A) Kaplan-Meier survival curves comparing beta-blocker users versus non-users, over a follow-up period of up to 6 years. (B) Kaplan-Meier survival curves after adjusting for covariates using Cox proportional-hazards regression.

Kaplan-Meier analysis demonstrated significantly better survival curve for patients on beta-blocker therapy, which was consistent throughout FU period (χ2=5.8, p=0.02) (figure 2A). However, when we adjusted for covariates associated with mortality (age, gender, hypertension, valvular heart disease, CKD and atrial fibrillation) using Cox proportional-hazards model, the Kaplan-Meier survival curves showed no statistically significant difference between the three groups (p=0.47) (figure 2B); thus, negating any association between beta-blockers and the survival of these patients.

**Figure 3:**
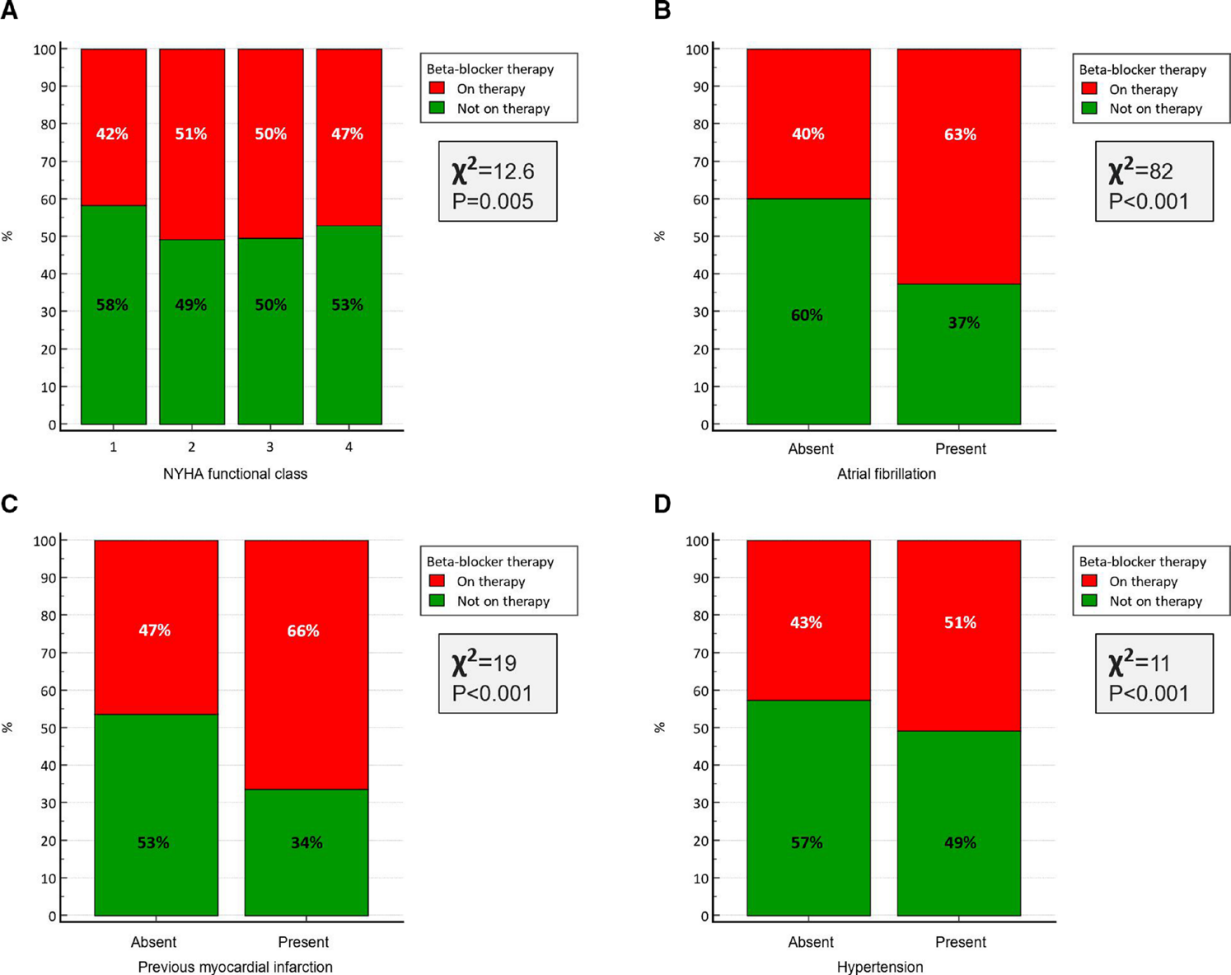
Clustered histogram demonstrating percentage of patients receiving beta-blocker or not receiving it. Patients have been characterised by: (A) NYHA functional class, (B) the presence or absence of atrial fibrillation, (C) the presence or absence of previous myocardial infarction, and (D) the presence or absence of hypertension. NYHA, New York Heart Association.

Not surprisingly, the use of beta-blockers was significantly higher in the management of patients with atrial fibrillation and ischaemic heart disease including previous myocardial infarction (table 4; figure 3). In addition, patients with hypertension and diabetes mellitus had significantly higher rate of use of beta-blockers (table 4, figure 3). It was also interesting to note that users of beta-blockers who had significantly higher mean NT-proBNP level (users: 1053±960 vs non-users: 846±62, p<0.001) were more likely to be symptomatic (figure 3) and were significantly younger than patients who were not on that therapy.

**Table 4:** Beta-blocker status according to cardiovascular risk factors for mortality

	No therapy	On therapy	P value
Age (years)	79±9	77±9	<0.001*
NT-proBNP (pg/mL)	849±628	1053±960	<0.001*
Gender (male)	391 (51%)	373 (49%)	0.43†
Hypertension	546 (49%)	564 (51%)	<0.001†
Diabetes	130 (42%)	177 (58%)	<0.001†
Ischaemic heart disease	145 (36%)	261 (64%)	<0.001†
Valvular heart disease	71 (56%)	56 (44%)	0.39
Previous MI	42 (34%)	83 (66%)	<0.001†
Chronic kidney disease	555 (53%)	482 (47%)	0.20
Atrial fibrillation	230 (37%)	384 (63%)	<0.001†

*ANOVA test with Tukey-Kramer post-hoc analysis.

†Χ^2^ test.

ANOVA, analysis of variance; MI, myocardial infarction; NT-proBNP, N-terminal pro-brain-type natriuretic peptide.

## Discussion

The present study sought to look at the association between common cardiovascular therapies and mortality among patients with symptoms suggestive of HF, raised NT-proBNP ≥400 pg/mL, but no evidence of HF on echocardiography. On univariate analysis, beta-blockers showed an association with survival, however that apparent association disappeared with multivariate Cox proportional-hazards regression that adjusted for all variables found in our previous report to be associated with mortality.^8^ None of the other cardiovascular therapeutic agents (diuretics, ACEi, MRA or digoxin) were shown to have any association with these patients’ survival.

One may legitimately pose the question of how the NT-proBNP levels are not significantly different between those in the cohort who died and those who have survived during FU, when we know that the rise of NT-proBNP is a marker of poorer prognosis even in the absence of HF? We note that the average NT-proBNP was less than 2000 pg/mL in both groups, and many had their NT-proBNP close to or even less than 900 pg/mL. We know that many current trials of HF stipulate that the admission threshold for patients with atrial fibrillation is 900 pg/mL. Indeed, 35% of the patients in the cohort had atrial fibrillation. We believe that the answer lies in the previously demonstrated fact by Kubanek *et al*^10^ who found the significant increase in all-cause mortality in patients with HF with reduced ejection fraction when the NT-proBNP is above 2530 pg/mL.

Why were these patients with no evidence of HF receiving beta-blockers (table 4, figure 3)? It was not surprising that beta-blockers’ use was significantly increased among patients with ischaemic heart disease including previous myocardial infarction and atrial fibrillation. In addition, the use of beta-blockers was significantly increased in patients with diabetes mellitus and hypertension. It is intriguing that users of beta-blockers had a higher mean NT-proBNP levels compared with non-users.

Although one may think it is counterintuitive, those symptomatic patients presenting to the HF clinic with raised NT-proBNP, but who had no evidence of HF on TTE, were more likely to survive during FU if they had atrial fibrillation or systemic hypertension.

It is of note, however, that in the cohort, only 63% of those with atrial fibrillation and between 64% and 66% of those with ischaemic heart disease were being treated with beta-blockers.

With regard to those with atrial fibrillation, it is not a surprise that 63% of them were on beta-blockers; however, we were surprised that 51% of those with systemic hypertension in this cohort were also on beta-blockers. While beta-blockers are not a first-line or second-line therapy for systemic hypertension, these can be used at any stage if there is another comorbidity that requires beta-blockers such as atrial fibrillation or ischaemic heart disease.^11^ Current guidelines of atrial fibrillation management emphasise the importance of controlling the ventricular rate with drugs including beta-blockers. A cohort study by Chao *et al* demonstrated that patients with atrial fibrillation receiving rate-control treatment with beta-blockers had a 24% lower risk of mortality compared with those with different or no rate-control treatments.^12^ In our cohort, those with atrial fibrillation may well have developed the symptoms and had the rise in the NT-proBNP purely because of the atrial fibrillation.

Our data do not provide us with the ability to answer the question of whether the apparently better survival in the patients of our cohort with atrial fibrillation and hypertension reflects an association based on the medical attention paid to treating these comorbidities or not.

A justifiable question is what were the causes of the raised NT-proBNP in the cohort if they did not have HF? We know from the comorbidities lists that the patients have, that 1037 patients (58%), 614 patients (35%) and 127 patients (7%) had, respectively, CKD II–IV, atrial fibrillation and valvular heart disease. These comorbidities could have contributed singularly or together when coexisting to the rise in the level of NT-proBNP without there being HF.

Another question is whether further tests should be routinely performed in a cohort of patients like ours, to ascertain the absence of HF? An observer could suspect that some of the patients in this cohort may have HF with preserved ejection fraction (HFpEF), which has progressively changing diagnostic criteria.^13^ We acknowledge this as a potential limitation. Indeed, longitudinal FU of the patients within our cohort may be justified on the basis of not only addressing their comorbidities, but also learning whether these patients go on to develop HF at a later stage.

Yang *et al* found, contrary to evidence-based guidelines, that treatment with beta-blockers was associated with significant improvement of survival rates in patients with HFpEF and atrial fibrillation (HR: 0.405, 95% CI: 0.233 to 0.701, p<0.001).^14^

In addition, and contrary to our study findings, Nielsen *et al* used three nationwide registries, and reported a potential clinical benefit of beta-blockers in patients with atrial fibrillation without HF.^15^ In that study, all-cause mortality among beta-blocker users was lower compared with non-users with a 1-year propensity-matched HR of 0.73 (95% CI: 0.71 to 0.76). Their study population included 205 174 patients with non-valvular atrial fibrillation; of those 164 433 were free from HF. The propensity-matched cohort of patients without HF with concomitant atrial fibrillation comprised 110 768 patients (67%). Therefore, there are published observations of large cohorts raising the spectre of a beneficial role of beta-blockers on the prognosis of patients with atrial fibrillation with no HF.

### Limitations

We have followed the guidelines in the diagnosis of HFpEF within the SHEAF registry, but we recognise the progressive changes to the criteria of that diagnosis, thus there remains some doubt of whether the stricter diagnostic criteria earlier in the life of the study might have labelled some patients as not having HF when the more recent criteria may have accepted their diagnosis being that of HFpEF.

The second limitation is the choice of the endpoint as all-cause mortality. Cardiovascular hospitalisation would have been another logical endpoint to look into. We chose all-cause mortality given its absolute incontrovertible nature and its availability to us. Looking at hospitalisation rate would have been possible, though it would have been very difficult in a service-based cohort to have accurate ascertainment of whether the hospitalisation was cardiovascular or not. We acknowledge this as a potentially major limitation to the study.

Another limitation is that we did not set out to check the degree of adherence to the variable important guidelines into the treatment of the various comorbidities the patients had such as hypertension, ischaemic heart disease, atrial fibrillation, diabetes mellitus or CKD. There is an opportunity for this to be considered in a future study.

A fourth limitation to this study is the fact we have not systematically undertaken to investigate the potential respiratory causes of the patients’ symptoms. Some of the specialists undertook pulmonary function tests, high-resolution CT scans and CT pulmonary angiograms. Others have either referred the patients to the respiratory clinic, while a third option was to ask the primary care physician to consider making such referral. As these were not options taken in a systematic manner, thus the data derived may not be reflective of the true incidence of the different respiratory causes of these patients’ symptoms. We have therefore refrained from reporting them in this manuscript.

## Conclusion

Patients with raised NT-proBNP but no HF on echocardiography may have lower mortality if they happen to have atrial fibrillation or hypertension. However, none of the cardiovascular medications they are taking appears to have an association with their survival during the 3-year FU.

## Data Availability

All data relevant to the study are included in the article. No further data are available.
